# Supplementary material for: How social evaluations shape trust in 45 types of scientists
Source: PLoS One. 2024 Apr 18;19(4):e0299621. doi: 10.1371/journal.pone.0299621 (PMC11025804; doi:10.1371/journal.pone.0299621)
Supplement: S1 File — (DOCX) [file pone.0299621.s001.docx]

**Pilot Studies**

With three pilot studies (*N* = 100 in each study), we aimed to develop a measure that reflects a behavioral consequence of trust. Our goal was to obtain a measure that would have convergent validity with trust (high correlations) and a distribution that would not be asymmetric (we aimed to avoid both floor and ceiling effects). In all three pilot studies, participants followed the same procedure, with the only difference in the wording of the influence granting task (IGT). Participants were presented with six scientific occupations (one from each of the six clusters of scientific occupations[6]) and asked to answer the same task for each scientific occupation. In order to eliminate any confounds, we made the task the same across all occupations. We aimed to construct a general task that could be then applied to all 45 occupations in the main study. The tasks, which were constant-sum questions (choices had to total 100) are described below (see Pilot Studies 1-3). Before completing the task, participants responded to two practice questions in order to familiarize themself with the instructions and answering format. After responding to a task for all six occupations (randomized order), participants filled out three measures of trust in the following order.

General trust was assessed by asking participants to respond to the question “How much do you trust [occupation]?” using a seven-point scale (1 = *do not trust at all*, 7 = *trust completely)* [1]. Next, they answered the four-item scale of trust which asked how much they trust or distrust [occupation] to: “create knowledge that is unbiased and accurate”, “create knowledge that is useful”, “advise government officials on policy?”, and “inform the public on important issues?”. Answers were given on a five-point scale from 1 = *completely distrust* to 5 = *completely trust* [31]. Finally, participants were asked to answer three items with the following question: “How much or little influence/control do you think [scientific occupation] should have: ‘over public policy’, ‘on the choices that other people make’ and ‘on the issues that matter to you’”. They responded using a five-point scale (1 = *none at all* to 5 = *a great deal*). After completing the three measures for one occupation, they completed the same measure for the next one, until responding to all six occupations. The order of occupations was randomized. In each study (N = 100), participants were recruited through Prolific (country filter: the US) and received £1.40 for their participation. The completion lasted around 10 minutes. The survey and data for all three pilot studies are provided on the project’s OSF page. An overview of sample demographics is given in Table S1.

| **S1 Table. Sample demographics of the pilot studies** | | | |
| --- | --- | --- | --- |
|  | **Gender** | **Age** | **Education** |
| *Pilot 1* | 50 men  50 women | M = 38.57  SD = 13.39 | Completed high/secondary school: 22  Undergraduate degree (BSc, BA): 46  I’m currently studying: 5  Graduate degree: 27 |
| *Pilot 2* | 51 men  46 women  3 other | M = 36.15  SD = 12.72 | Less than high/secondary school: 1  Completed high/secondary school: 28  Undergraduate degree (BSc, BA): 41  I’m currently studying: 12  Graduate degree: 18 |
| *Pilot 3* | 49 men  51 women | M = 42.89  SD = 13.04 | Completed high/secondary school: 36  Undergraduate degree (BSc, BA): 44  I’m currently studying: 7  Graduate degree: 13 |

Results of the pilot studies— i.e., correlations of the trust task with the three other measures of trust, as well as answering distribution—are given in S2 Table below. Based on the intercorrelations and means, we concluded that the phrasing in Pilot Study 2 was best (note that, for the main study, we added “must” in the last sentence).

***Pilot Study 1***

The phrasing of the task and the offered parties (all randomized except “scientific occupation” and “Other scientists” which were always the third and fourth option) in Pilot Study 1 was as follows:

Imagine there is a pressing societal problem in your country. You have the complete power to make a decision about how to solve the problem. This problem is very complex and, therefore, to solve it, the help and advice of various types of scientists would be useful. If you were to make a final decision, how strongly would you value the input of the following parties? Note that points sum up to 100.

Community leaders 0

Politicians 0

Citizens 0

[Scientific occupation] 0

Other scientists 0

Family members 0

Friends 0

Myself 0

Answers were given using a slider for each option, with a possible allocation of anywhere between 0 and 100 points per option. The points had to total 100.

***Pilot Study 2***

The phrasing of the task and the offered parties (all randomized) in Pilot Study 2 was as follows:

Imagine there is a pressing problem in your country that is affecting every citizen. You have the complete power to make a decision about how to solve the problem. This problem is very complex and, therefore, to solve it, the help and advice of various types of scientists would be useful. If you were to make a final decision, how strongly would you value the input of the following parties? Note that points sum up to 100.

Community leaders 0

Politicians 0

Citizens 0

[Scientific occupation] 0

Family members 0

Friends 0

Myself 0

Answers were given using a slider for each option, with a possible allocation of anywhere between 0 and 100 points per option. The points had to total 100.

***Pilot Study 3***

The phrasing of the task and the offered parties (all randomized except scientific occupation and Other scientists which were always third and fourth options) in Pilot Study 1 was as follows:

Imagine there is a pressing problem in your country that is affecting every citizen. You have the complete power to make a decision about how to solve the problem. This problem is very complex as it involves a combination of forces of nature, technical elements, impact on plants and animals, and societal aspects that need to be considered. Therefore, to solve the problem, the help and advice of various types of scientists would be useful. If you were to make a final decision, how strongly would you value the input of the following parties? Note that points must sum up to 100.

Community leaders 0

Politicians 0

Citizens 0

[Scientific occupation] 0

Family members 0

Friends 0

Myself 0

The answers were given using a slider for each option, with a possible allocation of anywhere between 0 and 100 points per option. The points had to total 100.

| **S2 Table. Pilot studies.** Descriptives for IGT and intercorrelations of four trust measures, for six different scientific occupations (Anthropologist, Biologist, Computer Scientist, Oceanographer, Physicist, Sociologist) in three pilot studies. Each trust measure is labeled with a number (1-4); distribution statistics (means, standard deviations, and medians) are given only for the IGT measure. Intercorrelations are given between all trust measures. There are three correlation coefficients (in order: Pearson’s r, Spearman’s rho, Kendall’s Tau B) because of the non-normal distribution. All the statistics presented in the table are given for each of the six occupations and each of the pilot studies. | | | | | | | | | | |
| --- | --- | --- | --- | --- | --- | --- | --- | --- | --- | --- |
|  | **Pilot 1** | | | **Pilot 2** | | | **Pilot 3** | | | |
| *Anthropologist* | | | | | | | | | | |
|  | 1. | 2. | 3. | 1. | 2. | 3. | 1. | 2. | 3. |  |
| 1. Task | *M* = 15.33  *SD* = 13.44  *Med* = 12 |  |  | *M* = 32.56  *SD* = 26.53  *Med* = 30 |  |  | *M* = 34.40  *SD* = 23.10  *Med* = 30 |  |  |  |
| 2. Overall trust | .193  .168  .132 |  |  | .332  .295  .228 |  |  | .283  .295  .230 |  |  |  |
| 3. Trust or distrust | .168  .197  .153 | .739  .596  .509 |  | .367  .328  .247 | .802  .763  .683 |  | .329  .343  .262 | .680  .726  .625 |  |  |
| 4. Influence/  control | .244  .240  .182 | .299  .224  .183 | .454  .430  .344 | .321  .364  .276 | .585  .511  .435 | .693  .588  .488 | .348  .467  .356 | .485  .493  .405 | .554  .515  .410 |  |
| *Biologist* | | | | | | | | | | |
|  | 1. | 2. | 3. | 1. | 2. | 3. | 1. | 2. | 3. |  |
| 1. Task | *M* = 16.59  *SD* = 14.27  *Med* = 15 |  |  | *M* = 40.03  *SD* = 27.53  *Med* = 36.5 |  |  | *M* = 45.90  *SD* = 22.61  *Med* = 42.5 |  |  |  |
| 2. Overall trust | .285  .275  .224 |  |  | .431  .435  .349 |  |  | .173  .205  .161 |  |  |  |
| 3. Trust or distrust | .253  .203  .161 | .630  .593  .520 |  | .481  .450  .343 | .750  .672  .588 |  | .212  .177  .130 | .744  .766  .688 |  |  |
| 4. Influence/  control | .231  .240  .175 | .287  .277  .236 | .498  .412  .330 | .416  .379  .275 | .507  .475  .400 | .583  .497  .402 | .185  .233  .161 | .595  .586  .496 | .610  .583  .474 |  |
| *Computer scientist* | | | | | | | | | | |
|  | 1. | 2. | 3. | 1. | 2. | 3. | 1. | 2. | 3. |  |
| 1. Task | *M* = 14.31  *SD* = 13.20  *Med* = 11.5 |  |  | *M* = 32.67  *SD* = 25.90  *Med* = 28 |  |  | *M* = 32.36  *SD* = 25.41  *Med* = 29 |  |  |  |
| 2. Overall trust | .217  .198  .161 |  |  | .286  .293  .225 |  |  | .221  .200  .147 |  |  |  |
| 3. Trust or distrust | .211  .195  .148 | .744  .557  .482 |  | .325  .263  .208 | .762  .764  .657 |  | .189  .179  .128 | .801  .779  .687 |  |  |
| 4. Influence/  control | .358  .361  .278 | .531  .485  .409 | .549  .484  .388 | .219  .192  .140 | .488  .449  .372 | .627  .595  .479 | .271  .278  .197 | .628  .620  .531 | .603  .587  .470 |  |
| *Oceanographer* | | | | | | | | | | |
|  | 1. | 2. | 3. | 1. | 2. | 3. | 1. | 2. | 3. |  |
| 1. Task | *M* = 13.06  *SD* = 14.16  *Med* = 10 |  |  | *M* = 28.88  *SD* = 25.86  *Med* = 28 |  |  | *M* = 36.57  *SD* = 25.44  *Med* = 32 |  |  |  |
| 2. Overall trust | .168  .130  .111 |  |  | .283  .209  .160 |  |  | .254  .323  .257 |  |  |  |
| 3. Trust or distrust | .086  .021  .018 | .639  .570  .497 |  | .301  .208  .153 | .730  .748  .640 |  | .232  .273  .203 | .512  .569  .502 |  |  |
| 4. Influence/  control | .326  .403  .311 | .481  .465  .386 | .454  .384  .311 | .406  .362  .264 | .506  .503  .404 | .698  .668  .538 | .280  .316  .228 | .530  .512  .426 | .537  .474  .386 |  |
| *Physicist* | | | | | | | | | | |
|  | 1. | 2. | 3. | 1. | 2. | 3. | 1. | 2. | 3. |  |
| 1. Task | *M* = 15.28  *SD* = 14.43  *Med* = 12 |  |  | *M* = 35.37  *SD* = 28.08  *Med* = 30 |  |  | *M* = 37.89  *SD* = 24.97  *Med* = 31 |  |  |  |
| 2. Overall trust | .084  .011  .013 |  |  | .336  .332  .265 |  |  | .307  .285  .225 |  |  |  |
| 3. Trust or distrust | .176  .158  .119 | .626  .605  .530 |  | .428  .332  .248 | .720  .709  .622 |  | .186  .222  .164 | .713  .799  .710 |  |  |
| 4. Influence/  control | .279  .256  .197 | .337  .300  .260 | .588  .546  .444 | .426  .372  .273 | .492  .512  .425 | .590  .548  .435 | .263  .293  .217 | .527  .528  .443 | .621  .590  .480 |  |
| *Sociologist* | | | | | | | | | | |
|  | 1. | 2. | 3. | 1. | 2. | 3. | 1. | 2. | 3. |  |
| 1. Task | *M* = 24.02  *SD* = 17.01  *Med* = 20 |  |  | *M* = 35.19  *SD* = 23.82  *Med* = 32 |  |  | *M* = 34.06  *SD* = 22.28  *Med* = 32 |  |  |  |
| 2. Overall trust | .352  .328  .255 |  |  | .330  .345  .260 |  |  | .292  .268  .209 |  |  |  |
| 3. Trust or distrust | .402  .381  .292 | .777  .708  .606 |  | .323  .326  .244 | .857  .805  .713 |  | .238  .184  .134 | .865  .821  .725 |  |  |
| 4. Influence/  control | .273  .252  .186 | .531  .488  .402 | .659  .599  .496 | .323  .365  .265 | .647  .597  .507 | .672  .643  .530 | .312  .344  .262 | .703  .688  .589 | .744  .735  .617 |  |
| *Note*. *M* = Mean, *SD* = Standard deviation, *Med* = Median. We do not mark significance levels because we were not interested in inference, but in estimating (correlation) size | | | | | | | | |  |  |


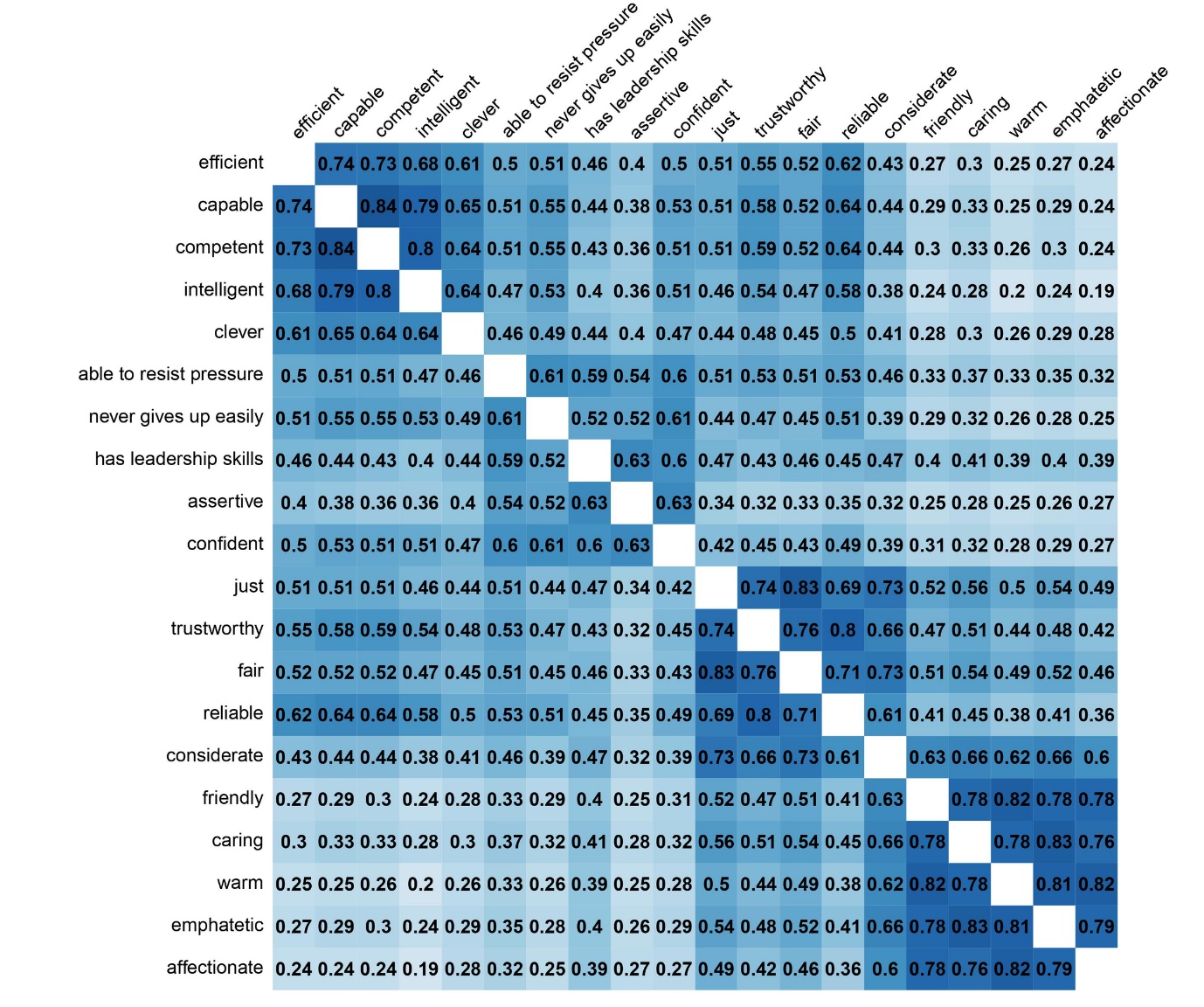


**S1 Fig**. Correlations between items measuring competence, assertiveness, morality, and warmth (each dimension was measured with five items). Stronger correlations are represented with stronger shades of blue.


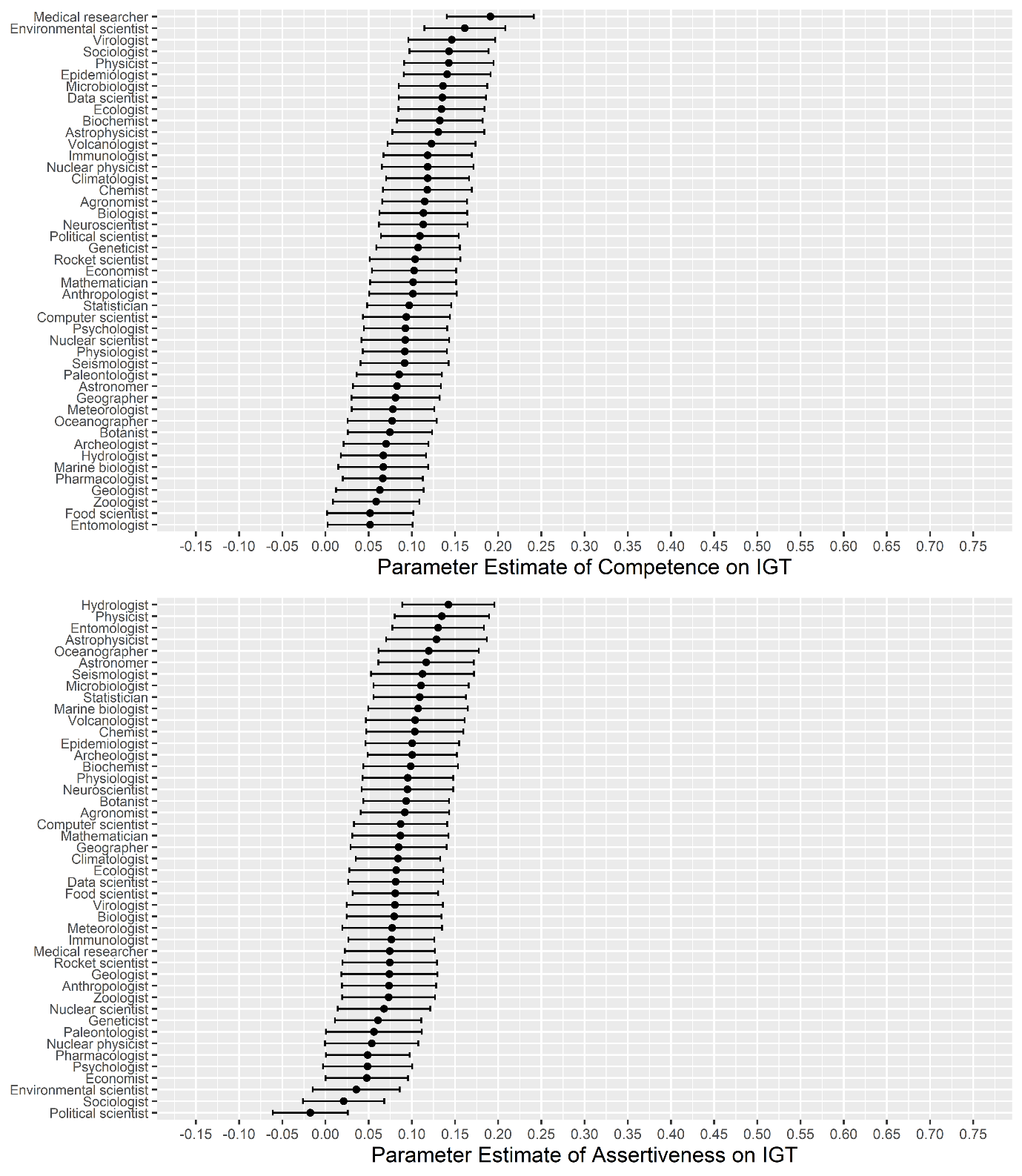


**S2 Fig.**


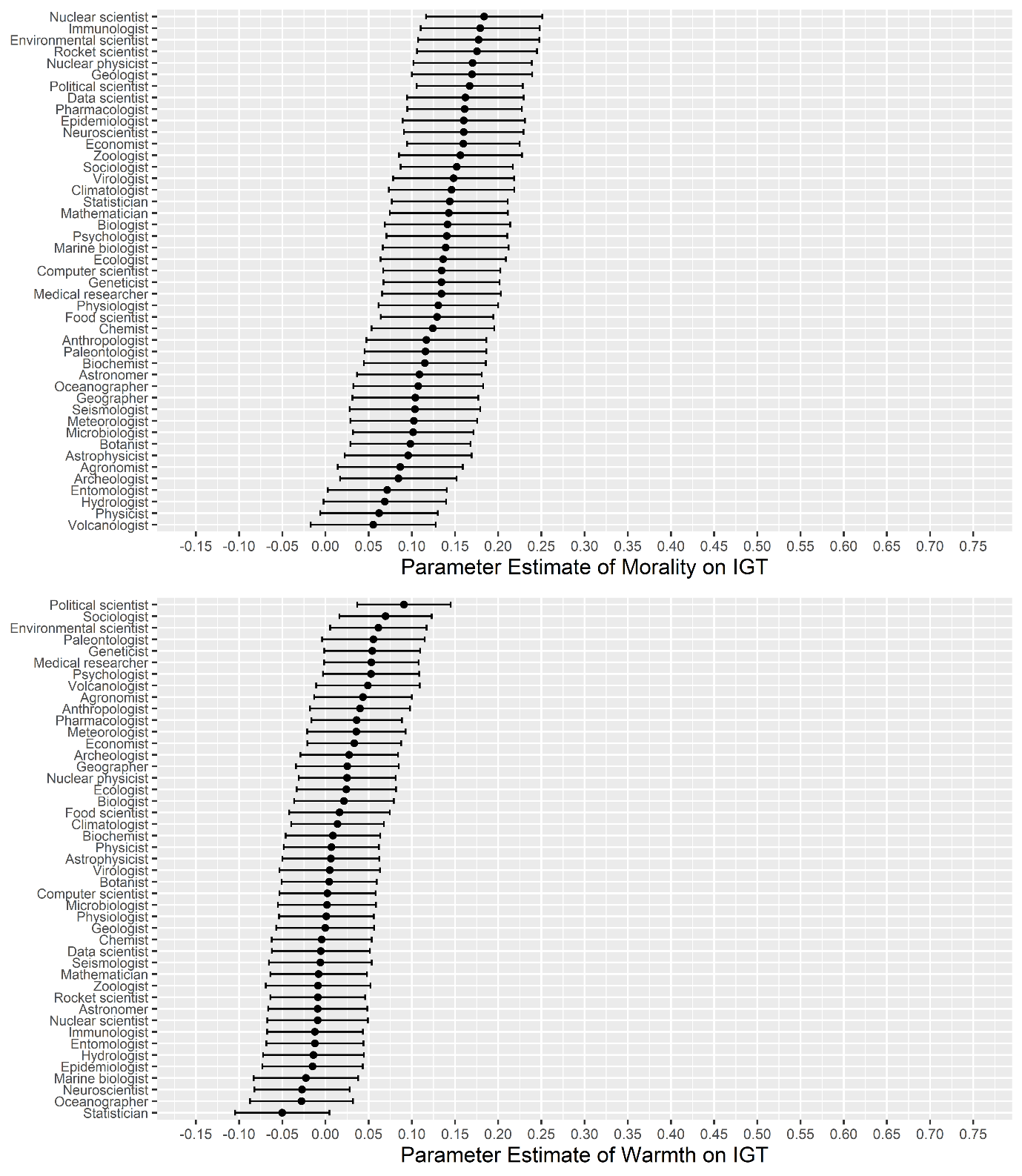


**S3 Fig**. **The effect of morality and warmth predicting influence granting.** The estimates (beta coefficients, with 95% confidence intervals) of morality and warmth predicting influence granting for all 45 occupations. All estimates were uniform across occupations.

| **Table S3.** Multilevel model (with a random intercept for participants and occupations) in which social evaluation measures predict trust and IGT (influence granting task) when “trustworthy” is excluded from the calculation of morality dimension | | | | |
| --- | --- | --- | --- | --- |
| Trust | | | IGT | |
|  | Fixed effects | | | |
|  | *β* (Standard error) | *t* value | *β* (Standard error) | *t* value |
| Competence | .22 (.01) | 23.44^***^ | .11 (.01) | 11.60^***^ |
| Assertiveness | .06 (.01) | 6.47^***^ | .08(.01) | 9.23^***^ |
| Morality | .39 (.01) | 38.94^***^ | .13 (.01) | 12.52^***^ |
| Warmth | .09 (.01) | 11.50^***^ | .02 (.01) | 2.082^*^ |
|  | Random effects | | | |
| τ_00 participant_ | .25 | | .63 | |
| τ_00 occupation_ | .07 | | .03 | |
| ICC | .53 | | .72 | |
| Marginal *R*^2^ /  Conditional *R*^2^ | .42/  .73 | | .08/  .74 | |
| *Note*. ^***^ *p* <.001, ^*^ *p* <.05, τ_00 =_ intercept variance, ICC = Intraclass correlation | | | | |

Variances below are calculated from a model that contains random effects for all social evaluations.

Variances for the effects of competence, assertiveness, morality and warmth on trust across occupations were respectively .001, .001, .007, .003.

Variances for the effects of competence, assertiveness, morality and warmth on IGT across occupations were respectively .001, .002, .002, .002.

Random indirect effects by occupation were also calculated from two regression models. In contrast to the averaged effects mediation model, we allowed random slopes of social evaluations on trust. We also included the random effect of trust on IGT. In this way, indirect effects were calculated from models in which all paths could vary across occupations.

| **Table S4**. Random indirect effects of competence, assertiveness, morality and warmth for each occupation (standardized) | | | | | |
| --- | --- | --- | --- | --- | --- |
| Occupation | Competence | Assertiveness | Morality | Warmth |  |
| Agronomist | 0.04 | 0.02 | 0.07 | 0.03 |  |
| Anthropologist | 0.04 | 0.01 | 0.10 | 0.01 |  |
| Archeologist | 0.03 | 0.01 | 0.05 | 0.02 |  |
| Astronomer | 0.05 | 0.02 | 0.09 | 0.01 |  |
| Astrophysicist | 0.05 | 0.02 | 0.08 | 0.02 |  |
| Biochemist | 0.05 | 0.01 | 0.12 | 0.02 |  |
| Biologist | 0.04 | 0.01 | 0.07 | 0.03 |  |
| Botanist | 0.04 | 0.01 | 0.07 | 0.01 |  |
| Chemist | 0.04 | 0.01 | 0.11 | 0.00 |  |
| Climatologist | 0.05 | 0.00 | 0.15 | 0.01 |  |
| Computer scientist | 0.04 | 0.01 | 0.10 | 0.02 |  |
| Data scientist | 0.05 | 0.02 | 0.13 | 0.02 |  |
| Ecologist | 0.05 | 0.01 | 0.12 | 0.02 |  |
| Economist | 0.04 | 0.00 | 0.15 | 0.03 |  |
| Entomologist | 0.04 | 0.01 | 0.07 | 0.01 |  |
| Environmental scientist | 0.04 | 0.00 | 0.13 | 0.01 |  |
| Epidemiologist | 0.06 | 0.02 | 0.13 | 0.02 |  |
| Food scientist | 0.03 | 0.01 | 0.09 | 0.02 |  |
| Geneticist | 0.04 | 0.01 | 0.09 | 0.02 |  |
| Geographer | 0.04 | 0.02 | 0.04 | 0.02 |  |
| Geologist | 0.04 | 0.01 | 0.09 | 0.02 |  |
| Hydrologist | 0.05 | 0.02 | 0.09 | 0.02 |  |
| Immunologist | 0.05 | 0.01 | 0.11 | 0.02 |  |
| Marine biologist | 0.04 | 0.01 | 0.08 | 0.01 |  |
| Mathematician | 0.05 | 0.01 | 0.11 | 0.01 |  |
| Medical researcher | 0.05 | 0.01 | 0.12 | 0.03 |  |
| Meteorologist | 0.03 | 0.00 | 0.10 | 0.00 |  |
| Microbiologist | 0.06 | 0.02 | 0.10 | 0.02 |  |
| Neuroscientist | 0.05 | 0.01 | 0.10 | 0.01 |  |
| Nuclear physicist | 0.04 | 0.01 | 0.11 | 0.01 |  |
| Nuclear scientist | 0.04 | 0.01 | 0.12 | 0.02 |  |
| Oceanographer | 0.04 | 0.02 | 0.07 | 0.01 |  |
| Paleontologist | 0.03 | 0.01 | 0.05 | 0.02 |  |
| Pharmacologist | 0.03 | 0.00 | 0.08 | 0.04 |  |
| Physicist | 0.05 | 0.02 | 0.10 | 0.02 |  |
| Physiologist | 0.04 | 0.01 | 0.09 | 0.02 |  |
| Political scientist | 0.03 | 0.01 | 0.12 | 0.04 |  |
| Psychologist | 0.04 | 0.00 | 0.13 | 0.02 |  |
| Rocket scientist | 0.05 | 0.01 | 0.10 | 0.02 |  |
| Seismologist | 0.04 | 0.02 | 0.07 | 0.01 |  |
| Sociologist | 0.03 | 0.01 | 0.10 | 0.02 |  |
| Statistician | 0.05 | 0.01 | 0.13 | 0.01 |  |
| Virologist | 0.05 | 0.01 | 0.13 | 0.02 |  |
| Volcanologist | 0.04 | 0.02 | 0.08 | 0.01 |  |
| Zoologist | 0.03 | 0.01 | 0.06 | 0.01 |  |


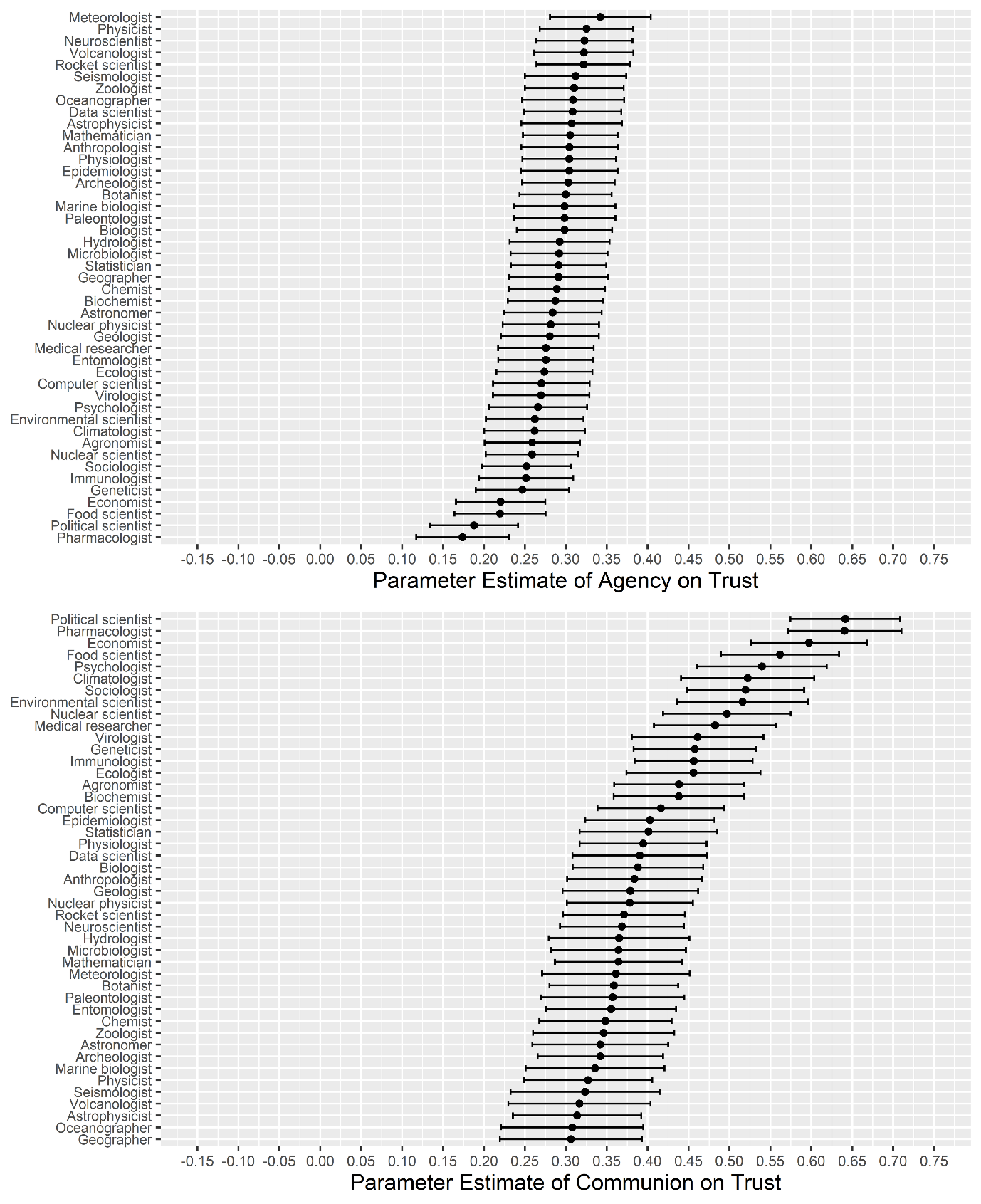


**S4 Fig**. The estimates of big two social evaluations (agency and communion) predicting trust for all 45 occupations. The estimates represent beta coefficients, with 95% confidence intervals.





**S5 Fig. The effect of the big two (agency and communion) predicting influence granting via trust**. The model is averaged across occupations and participants with random intercepts for both factors. Trust partially mediated the effects of agency and communion (respectively 35% and 82% proportion of mediated effect). Significant paths are marked with three asterisks (^***^p < .001). Direct effects are given within brackets.
