# Supplementary material for: How social evaluations shape trust in 45 types of scientists
Source: PLoS One. 2024 Apr 18;19(4):e0299621. doi: 10.1371/journal.pone.0299621 (PMC11025804; doi:10.1371/journal.pone.0299621)
Supplement: Attachment — Submitted filename: Reviewer response letter.docx [file pone.0299621.s003.docx]

Dear Dr. Claudia Noemi González Brambila,

We would like to thank the reviewer for the feedback and thank you for inviting us to submit a revision. As last time, we did our best to address the reviewer’s points and revised the manuscript where needed. We are providing below responses to each point and show how we changed the manuscript. Our responses in the letter are provided in blue, while the changes in the manuscript are highlighted in yellow.

We hope that this revision addresses the comments raised sufficiently. We look forward to hearing from you again.

With best regards,

Reviewer #1: I guess it's good; It's really interesting data and is well presented. I just still wish you didn't use a direct measure of trust OR called out the fact it's probably a problematic and then compared it to your better measure.

1. The argument that a direct, single-measure trust measures is validated isn't really clear.

2. The argument that it's valid because it's used a lot by others (including non-academic researchers) isn't very compelling.

We now rephrased this whole part to make it clearer and we also added arguments that the one-item measure is a proper substitute for a multi-item measure ([Castro et al., 2023](https://www.mdpi.com/2076-328X/13/9/786)) and it predicts a range of behavior/intentions (e.g., intention to comply with protective measures in COVID, [Algan et al., 2021](https://www.pnas.org/doi/abs/10.1073/pnas.2108576118); [Han, 2022](https://www.cambridge.org/core/journals/epidemiology-and-infection/article/trust-in-the-scientific-research-community-predicts-intent-to-comply-with-covid19-prevention-measures-an-analysis-of-a-largescale-international-survey-dataset/EF3CC812C471EC86C4D7012A6587F345); pro-environmental behavior; [Cologna et al., 2022](https://www.sciencedirect.com/science/article/pii/S0272494421001948#bib5)). This part now reads as follows (p. 8):

After rating an occupation on the social evaluation scales, trust was measured using the following item: “How much do you trust [occupation]?” (1 = *do not trust at all* to 7 = *trust completely*) [1]. We opted for this approach to measure trust for several reasons. First, various large-scale research studies have used this operationalization [1,3], which predicts a range of behaviors[28,29] and allows participants to construct their own meaning of trust, rather than researchers imposing theirs. Second, a one-item measure has been shown to function well as a substitute for a multi-item measure of trust[30]. Third, our pilot studies, which included both one-item and a more specific four-item[31] measures of trust, indicate that these correlate strongly (median Pearson’s *r* = .74 across different occupations and pilot studies). Finally, a single-item measure is cost-effective, which was of importance given the large number of participants and occupations in the study.

3. Your argument that's common method variance is creating high correlations is probably partly correct but the more likely problem is that you're comparing at set of specific attitudes to a general attitude rather than an unclear measure that's probably capturing a bit of behavior and a bit of attitude. It's like you ask, how much do you like rap, jazz, R&B, rock, etc. and then correlated it with 'how much do you like music' (vs. would you be willing to go to a show/buy a song). Of course the attitude-attitude (or belief-belief) is going to be higher than the belief-behavioral willingness.

This is a good point. We now added this as the first reason: “We believe these differences emerged because social evaluations more closely matched trust perceptions in that both can be seen as attitudinal measures, whereas the IGT is more a behavioral measure.” (p. 21)

4. Your argument that Fiske's BIAS model doesn't include a behavior isn't quite right. What are active/passive facilitation and harm if not behaviors?

This relates to a point raised in the previous letter. Previously, the reviewer noted that SCM/BIAS model has a good set of outcomes that we could have used. However, we argued that none of these behaviors relate to trust, and not that they do not relate to behavior (they *are* behaviors, just not related to trust). Indeed, the BIAS map (Cuddy, Fiske, & Glick, 2007), includes the following factors with respective items:

Active facilitation: help, protect

Active harm: fight, attack

Passive facilitation: cooperate with, associate with

Passive harm: exclude, demean

Although these behaviors are interesting, they unfortunately do not relate to trust, which is why we could not use them in this work.

5. Your comment that Besley et al 2021 doesn't include a measure of trust is incorrect. Table 2 and Table 5 include both a direct measure of trust and a trust-as-vulnerable-behavior measures. As with your study, it looks like there's a higher correlation between the direct measure (i.e., attitude to attitude) and the trustworthiness measures than the vulnerability-trustworthiness measures.

In the previous version, we wrote the following: “Yet, the three-component model of trust has also shown to be applicable within the context of scientists: Hendriks and colleagues (2015) found that scientists were evaluated on these three dimensions[23]. More recently, it has been suggested that the factor of “openness” should be added as a fourth component of trust in scientists[9]. **However, neither study tested how these dimensions contribute to trust, due to high intercorrelations between components**”

We do agree that we did not clearly state what we had in mind. We meant that the *relative contribution* of predictors has not been tested. We now rephrased the bolded part to: “**However, due to high intercorrelations between components, neither study tested the relative contribution of these dimensions to trust**.” (p. 6). We apologize for the confusion.

6. I still think you should point out that warmth and assertiveness (perceptions about how people BEHAVE) are qualitatively different than morality and competence (perceptions about how people ARE), unless I missed it.

This is a good suggestion, which we now added to the discussion (p.20): “It is also noteworthy that competence (vs. assertiveness) and morality (vs. warmth) as strongest predictors are more stable and central to one’s character as they refer to perception how people are (vs. how they behave/seem) (Abele & Hauke, 2020)”

Also, please note that we removed footnote 3 and integrated it with text (which comes before the part we now added)
